# Lassa virus protein–protein interactions as mediators of Lassa fever pathogenesis

**DOI:** 10.1186/s12985-025-02669-y

**Published:** 2025-02-28

**Authors:** Sharon Jan, Kruttika S. Phadke, Victor L. Lam, Steven S. Branda, Dylan M. Johnson

**Affiliations:** 1 Department of Biotechnology and Bioengineering, Sandia National Laboratories, Livermore, CA 94550 USA; 2 Department of Systems Biology, Sandia National Laboratories, Livermore, CA 94550 USA

**Keywords:** Lassa virus, Lassa fever pathogenesis, Protein–protein interactions, Host-virus interactions, Arenavirus, Emerging RNA virus, Hemorrhagic fever virus

## Abstract

Viral hemorrhagic Lassa fever (LF), caused by Lassa virus (LASV), is a significant public health concern endemic in West Africa with high morbidity and mortality rates, limited treatment options, and potential for international spread. Despite advances in interrogating its epidemiology and clinical manifestations, the molecular mechanisms driving pathogenesis of LASV and other arenaviruses remain incompletely understood. This review synthesizes current knowledge regarding the role of LASV host-virus interactions in mediating the pathogenesis of LF, with emphasis on interactions between viral and host proteins. Through investigation of these critical protein–protein interactions, we identify potential therapeutic targets and discuss their implications for development of medical countermeasures including antiviral drugs. This review provides an update in recent literature of significant LASV host-virus interactions important in informing the development of targeted therapies and improving clinical outcomes for LF patients. Knowledge gaps are highlighted as opportunities for future research efforts that would advance the field of LASV and arenavirus pathogenesis.

## Introduction

The family Arenaviridae contains five genera: *Mammarenavirus, Reptarenavirus, Hartmanivirus, Antennavirus,* and *Innmovirus* [1, 2]*.* However, only *Mammarenaviruses* infect mammals and are clinically relevant [3]. *Reptarenaviruses* infect the Boidae family of snakes and cause inclusion body disease, while naturally occurring *Hartmaniviruses* have been identified only in hosts co-infected with *Reptarenaviruses* [1, 4–6]. *Antennaviruses* infect and cause disease in frogfish and salmon [7, 8]. The host species of the recently described *Innmoviruses* has yet to be discovered; however, given the genetic similarity to *Antennaviruses* and its isolation from a river bed, the *Innmovirus* host is likely an aquatic species [9]. Within *Mammarenavirus*, the viral species are split into two categories: Old World (OW) and New World (NW) viruses. OW arenaviruses include the prototypic lymphocytic choriomeningitis virus (LCMV) as well as Lassa virus (LASV), the causative agent of Lassa hemorrhagic Fever (LF) [10]. NW arenaviruses include several clinically relevant viruses that cause hemorrhagic fever disease: Junín, Machupo, Guanarito, Sabia, and Chapare viruses [11]. Hemorrhagic fever causing NW arenaviruses exhibit significant genetic diversity. Exploring the structural and functional similarities between OW and NW arenaviruses can deepen insight into arenavirus pathogenicity and transmission. It is important to recognize that much of the current knowledge about arenavirus biology is derived from studies including LCMV and other hemorrhagic NW arenaviruses. As such, caution is warranted when extrapolating findings from these studies across pathogenic arenaviruses including LASV. LASV is prevalent in West Africa, particularly in Nigeria, Guinea, Sierra Leone, and Liberia where it is enzootic in multimammate rodents of the species *Mastomys natalensis* which become persistently infected when exposed to the virus early in life, but remain asymptomatic [10, 12]. Estimates based on human seroprevalence suggest that there are up to 500,000 annual infections with 20–50% resulting in symptomatic disease. However, far fewer meet the case definition criteria, and LF typically has an overall case fatality rate (CFR) of less than 2% [13]. On the other hand, the CFR in hospitalized patients can be markedly higher; for example, recent outbreaks in Nigeria have had CFRs over 25% [14, 15]. Survivors of LF often develop sudden onset sensorineural hearing loss (SNHL), creating additional public health burden [16]. The vast genetic diversity within LASV strains, along with under-sampling of sub-clinical cases, makes it difficult to correlate LASV genotypes with pathogenicity [17]. Comprehensive reviews of the clinical course of LF and ongoing efforts to develop medical countermeasures [18] and of animal models of LF [19], have been published recently. This review updates the literature with recent discoveries in host-virus interactions that occur during LASV infection and their impact on LF pathogenesis, paying particular attention to protein–protein interactions (PPI), but also considering protein-viral RNA interactions.

## RING-finger matrix protein Z

**Fig. 1:**
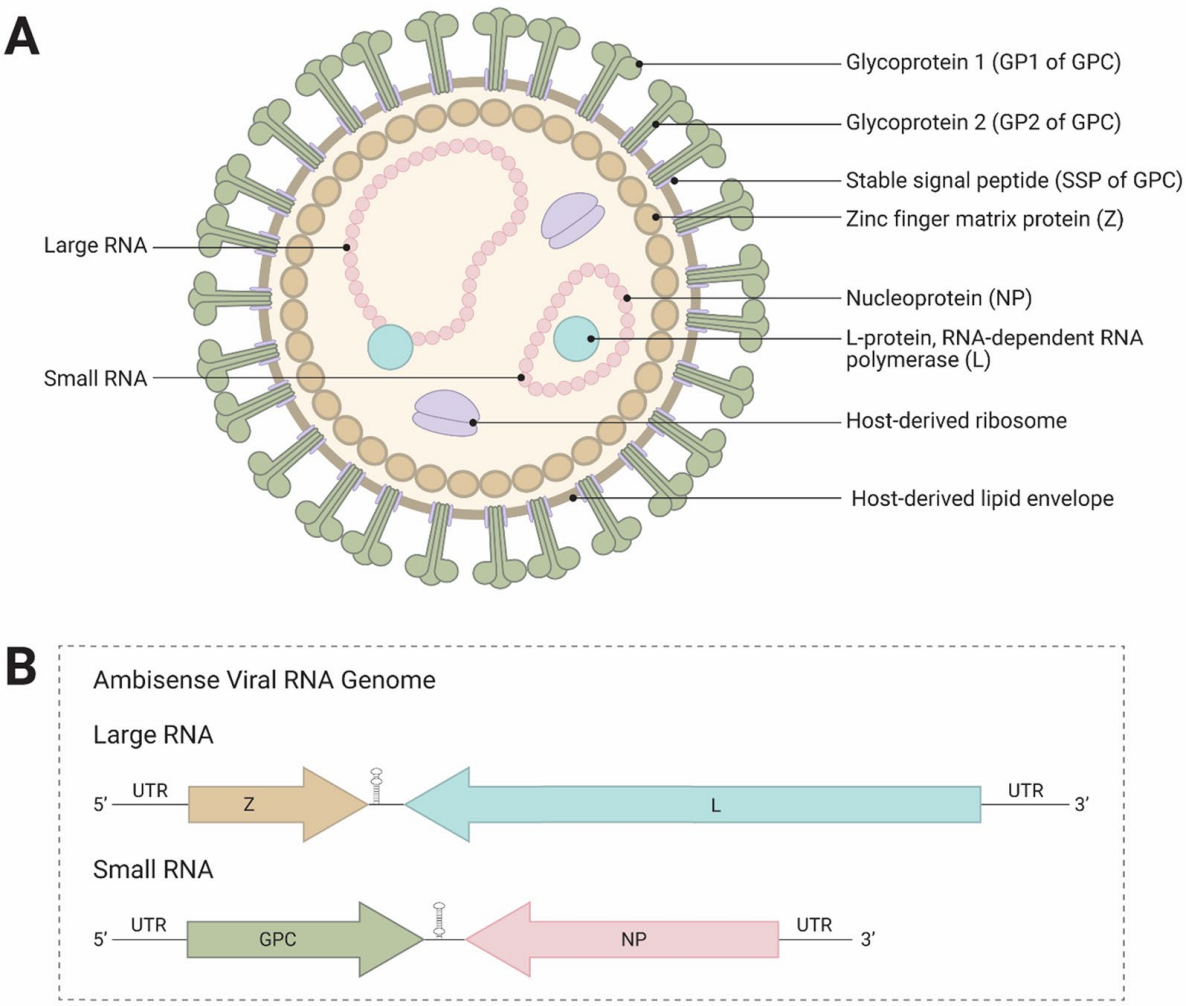
Arenavirus structure and genome organization. **A** Arenaviruses are 90–110 nm in diameter and have a host-derived lipid envelope expressing glycoprotein complex (GPC) spikes containing glycoprotein 1 (GP1), glycoprotein 2 (GP2), and stable signal peptide (SSP) on the surface. Inside the membrane is a layer containing the zinc finger matrix protein (Z) and viral RNA encapsulated by nucleoprotein (NP). NP with the viral RNA acts as a scaffold for the ribonucleoprotein complex involving the L protein RNA-dependent RNA polymerase (RdRP). Viral RNA is split into two species, a large and small RNA. **B** Arenavirus ambisense RNA encodes for four proteins. The large RNA encodes for Z and L, while the small RNA encodes for GPC and NP. Both segments are flanked by 5’ and 3’ untranslated regions (UTRs) and have structurally distinct intergenic regions (IGRs) which are important in terminating transcription

LASV has a bi-segmented, ambisense RNA genome. The 7.3 kBp large (L) segment encodes a RING-finger matrix protein (Z) in positive sense, and an RNA-dependent RNA polymerase (RdRP) L protein [20] in antisense. The small (S) segment is about 3.4 kBp and encodes two genes: glycoprotein precursor (GPC) in positive sense, and nucleoprotein (NP) in antisense (Fig. 1). Following viral entry into the cell, the L protein packaged inside the virion transcribes NP and L mRNA from the packaged viral genome, and also synthesizes antigenomes. The antigenomes then serve as templates for transcribing GPC and Z mRNA and replicating the viral genome.

**Fig. 2:**
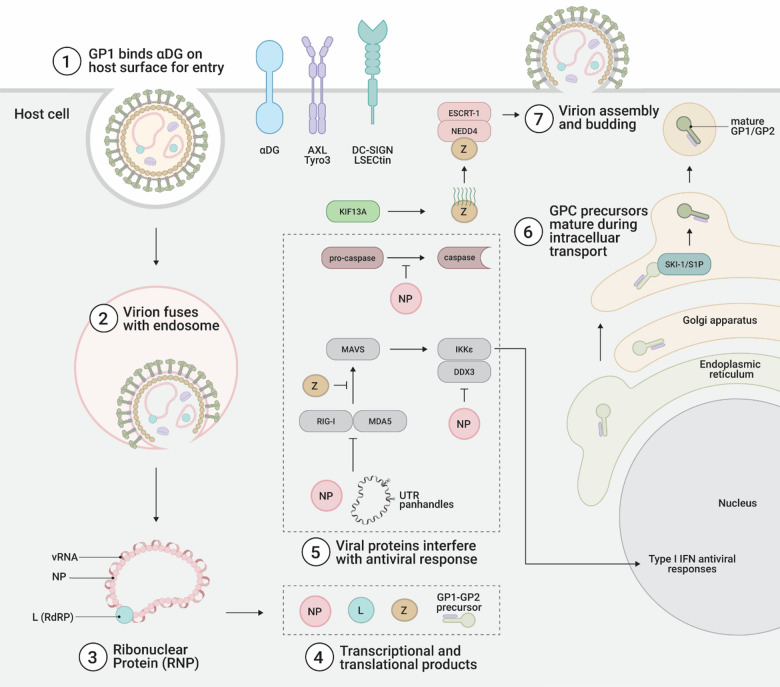
Overview of protein interactions during the LASV arenavirus life cycle in the host cell. **1** GP1 present on the viral surface binds α-dystroglycan for primary attachment. Receptors AXL, Tyro3, DC-SIGN, and LSECtin are also involved in interanalizing the LASV virion. Glycosylation of GP1 hinders neutralizing antibody binding. **2** pH-dependent GP1 binding to LAMP1 in the late endosome precedes GP2-mediated fusion. GP2 causes fusion at the endosomal membrane and deposition of viral contents into the host cytoplasm. **3** Once in the cytoplasm, RNP comprised of L protein (RdRP), NP, and vRNA initiate transcription and replication of the viral genome. Replication effiency correlates well to pathogenicity. **4** Viral transcriptional and translational products include NP, L protein, Z, and the GP1-GP2 precursor. **5** Viral proteins interfere with the host antiviral response and aid in immune evasion and supression. NP degrades dsRNA, preventing RIG-I and MDA5 recognition. 5’ and 3’ UTRs form panhandle structures with single nucleotide mismatches, inhibiting RIG-I and MDA5. Z inhibits RIG-I and MAVS association. NP also modulates IKKε and DDX3 activity, preventing establishment of an antiviral state in the host cell. NP has decoy caspace cleavage sites which decrease apoptosis. **6** GPC maturation from the GP1-GP2 precursors and transport occur in the endoplasmic reticulum and Golgi apparatus. SSP is co-translationally cleaved from GPC, and SKI-1/S1P processes GPC for maturation and formation of virions and fusions. **7** Virions assemble at the surface of the host cell and bud off from the plasma membrane with a host-derived lipid envelope. Z late domains PTAP and PPPY interact with ESCRT-1 and NEDD4 to mediate multivesicular budding

All four of the proteins encoded by LASV are multifunctional and contribute to viral pathogenesis. The lifecycle pathway of LASV in the host cell is depicted, highlighting several important interactions that are expanded on in the remainder of this manuscript (Fig. 2). The LASV Z protein forms a shell under the envelope of LASV virions, and mediates budding, but it also interacts with several host proteins [21]. Z protein strongly associates with the membranes of infected cells and mediates budding of nascent virions, with expression of LASV Z protein alone in cell culture being sufficient for the budding of viral like particles (VLPs) that contain Z [22]. Self-assembly of VLPs appears to be an ordered event that is dependent on the RING zinc-binding site II of Z [23].

**Fig. 3:**
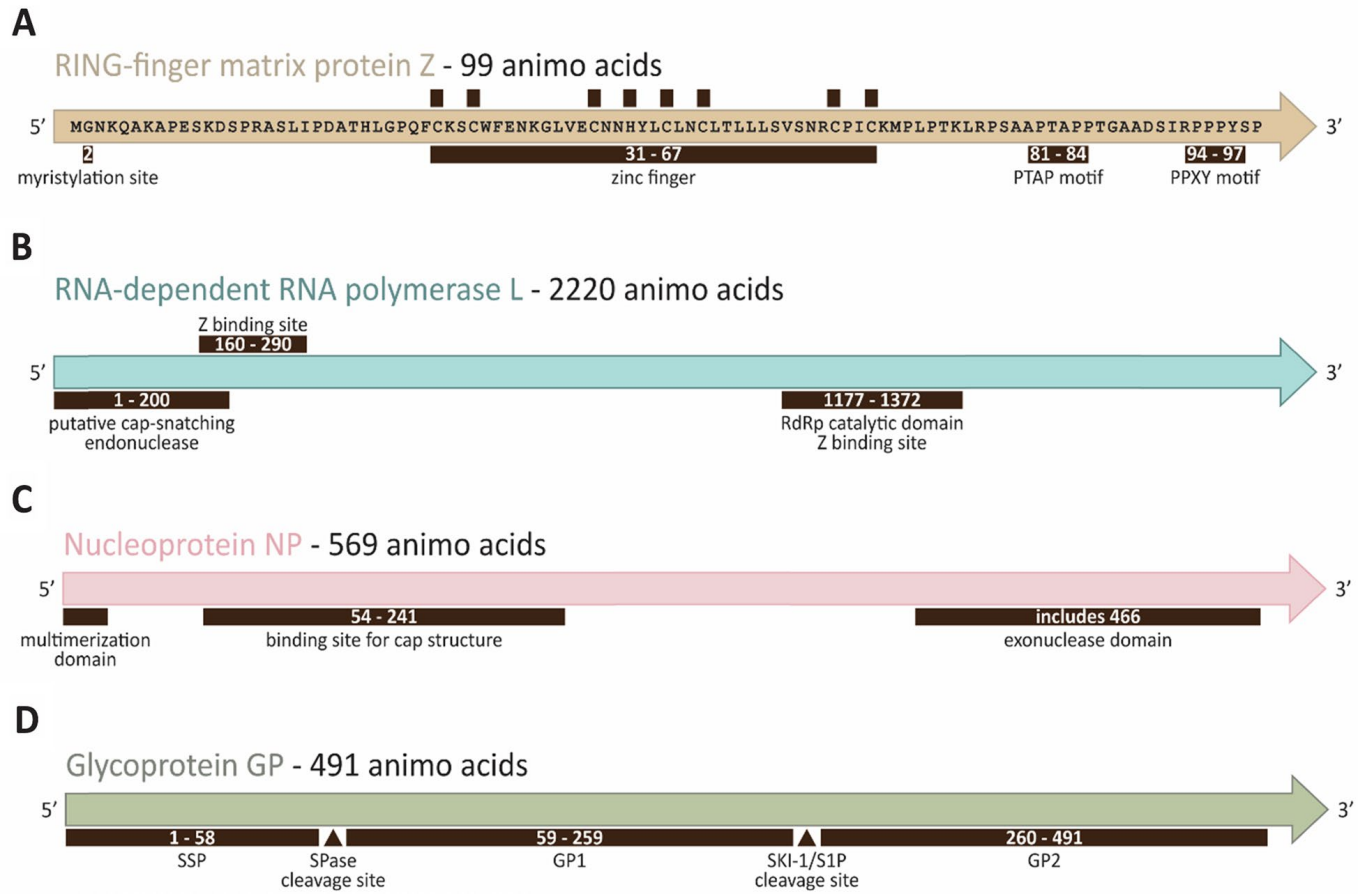
Features and domains of the LASV genes. **A** RING-finger matrix protein Z has a myristylation site at glycine 2, a zinc finger domain in the middle, and PTAP and PPXY motifs at the C-terminus. **B** RNA-dependent RNA polymerase (RdRP) L has a cap-snatching endonuclease at the N-terminus and an RdRP catalytic domain. **C** Nucleoprotein NP consists of a multimeraization domain at the N-terminus and an exonuclease domain at the C-terminus. **D** Glycoprotein GP is divided into SSP, GP1, and GP2 with accompanying cleavage sites

LASV Z protein is co-translationally modified by myristylation of the highly-conserved position 2 glycine near the N-terminus, which mediates interaction between Z, GP2 (the transmembrane domain of the GPC cleavage product), and the host cell membrane [24, 25] (Fig. 3A). Mutation of the myristylation site strongly inhibits the association of LASV Z with cell membranes and decreases viral budding [25, 26]. Additionally, myristylation is required for Z and GP2 interaction, leading to a model in which myristylated Z associates with both the host cell membrane (which becomes the viral envelope) and the membrane-anchored cytoplasmic tail of GP2 to mediate budding, and to form a shell of sub-membrane matrix protein in the mature virion [24, 26, 27].

The Z protein consists of C (cysteine) and H (histidine) residues forming the RING finger domains which coordinate the binding of two zinc ions (Fig. 3A). Late domains near the C terminus include PTAP and PPPY (P, proline; Y, tyrosine; T, threonine; A, alanine) motifs that historically play a role in viral release [26, 28]. Conserved proline-rich PPxY late-domain motifs present on the LASV Z protein play an essential role in virus egress by recruiting host proteins such as NEDD4 and YAP through interaction with their WW domains to mediate budding [29]. Additionally, two motifs in the C-terminal late domain of Z, PTAP and PPPY, have been shown to be critical for budding, such that mutating or deleting these motifs reduces budding by about 50% or 90%, respectively [22]. It is likely that PTAP interacts with the Tsg101 domain of host protein Endosomal Sorting Complex Required for Transportation-1 (ESCRT-1), whereas PPPY recruits NEDD4, with both interactions mediating budding through the multivesicular body pathway [21, 30–32]. In contrast, binding of LASV Z protein to WWOX, a host cell tumor suppressor regulating the Hippo pathway, apoptosis, and other physiologically critical cellular pathways, inhibits viral egress via its WW1 domain [29]. WWOX is hypothesized to competitively inhibit egress by relocating Z away from the budding site and into the nucleus. There is evidence that the interaction of LASV Z with kinesin family member 13A (KIF13A) contributes to microtubule mediated transport of Z to the periphery of infected cells [33, 34].

Arenavirus Z proteins are also known to interact with the viral ribonuclear protein complex (RNP), a structure formed by the association of L and NP proteins with viral RNA (vRNA), to inhibit viral replication based on studies with LCMV and Tacaribe virus (TCRV), a non-pathogenic NW virus [35, 36]. The RING-finger domain of Z interacts directly in a pH-dependent manner with the carboxy terminus of NP, which promotes the recruitment and incorporation of RNPs into virions [37–39]. The Z protein structure determined by the RING domain is necessary but not sufficient to mediate the Z mediated interference with the LCMV RNP [40]. This is also true for LASV where overexpression of LASV-Z locks the viral RdRP-L in a promotor-bound configuration that prevents translation [41, 42]. The L-binding domain on Z protein, made up of three points of contact on the central helix separating its two zinc finger motifs, allosterically regulates RdRP-L conformation and inactivates RdRP transcriptional activity [43]. Additionally, Z binding blocks nucleotide triphosphate access to L, preventing vRNA elongation [44]. Other conserved residues on Z outside of its RING and late domains have also been shown to modulate RNP activity [45]. Z-mediated RNP interference could play a role in balancing viral replication and the host response to allow the establishment of persistence in rodent hosts [42]. It is also possible that this interaction serves a purpose similar to that of DNA virus late proteins, which enact a switch in infected cells from viral replication to virion assembly and budding.

Z also plays a pivotal role in modulating the host's innate immune response by inhibiting the production of type I interferon (IFN) and downregulating the expression of IFN-stimulated genes (ISGs). Z is targeted by tetherin, a host interferon response protein that limits the release of mature LASV virions from infected cells [46, 47]. However, there are conflicting reports on the ability of Z proteins from Old World arenaviruses to antagonize the host IFN system by interfering with the association of retinoic acid-inducible gene I (RIG-I) and Mitochondrial AntiViral Signaling (MAVS) to diminish the IFN response to cellular vRNA in a similar fashion to New World arenavirus Z [48, 49]. LASV Z, but not non-pathogenic NW Pichinde virus (PICV) Z, subverts host RIG-I signaling to reduce IFN mediated host antiviral responses, which corroborates Z proteins as a pathogenic determinant [50]. The inhibition of human and rodent reservoir Mastomys RIG-I is a conserved host interaction for LASV and LCMV, although this interaction displays some variability which is difficult to correlate directly with pathogenicity [51].

Promyelocytic leukemia protein (PML), widely studied as a tumor suppressor protein, plays a role in the regulation of many host cellular processes including apoptosis, antiviral responses, cell cycle arrest, and inflammation [52]. PML binds directly to arenavirus Z [53]. For LCMV Z protein, this interaction precipitates the redistribution of PML from nuclear bodies to the cytoplasm, leading to a pro-apoptotic state [54]. Additionally, PML is downregulated at the protein level during LCMV infection [55]. While PML knockout increases viral replication, overexpression of PML does not affect the replication of LCMV or LASV, suggesting a role for PML as a mediator, rather than a direct effector, of antiviral responses [56, 57]. PML and LCMV Z both individually bind the host proline-rich homeodomain protein (PRH) through their RING-domains [58]. PRH is downregulated during pathogenic, but not non-pathogenic, LCMV infection; and this leads to suppression of the PRH hepatic antiproliferative effect, thereby promoting viral infection of the liver [59]. Although PML is downregulated and relocated during LCMV infection, the ribosomal P proteins which normally co-localize with PML are not redistributed [55]. Instead, Z binds to the nuclear fraction of P0, perhaps for its nuclear processing capabilities, which also precipitates the incorporation of host ribosomes into virions [55].

The complex formed by PML and Z also associates directly with eIF4E, through their respective RING domains, to directly antagonize mRNA cap binding, thereby hindering both viral and cellular translation [60]. Arenavirus L uses a cap-snatching mechanism to initiate transcription [61]. The PML-Z-eIF4E complex may play a role in accessing cellular mRNA to enhance cap-snatching, although this has not been demonstrated experimentally [62]. However, the RNP alone can replicate within cells as exhibited in a minigenome system, indicating that LCMV cap-snatching can occur in the absence of Z [35]. The endonuclease function of LASV L is not active in a cell-free system, which suggests that cap snatching is dependent on activation through interaction with NP, cis-acting factors, and/or host factors [63]. The downstream implications of the PML-Z-eIF4E interaction during arenavirus infection have yet to be fully explored [64]. Essentially, host PML exhibits antiviral functions via downstream signaling, and development of nuclear bodies is indicative of sequestration of viral particles. Interaction with viral Z results in downregulation of antiviral activity and nuclear bodies with the redistribution of PML, eventually leading to repressed cell growth and apoptosis. The PML-Z-eIF4E complex also downregulates translation by hindering eIF4E’s ability to transport mRNA, which ultimately impacts viral production and host defense.

Autophagy appears to play a key role in the generation of infectious LASV particles, although the mechanism has yet to be described [65]. For both LASV and the closely-related but non-pathogenic OW Mopeia virus (MOPV), the Z protein interacts with autophagy receptors through their calcium-binding and coiled-coil domain 3 (TAX1BP1) or calcium-binding and coiled-coil domain 2 (NDP54) [65]. Similarly, infection with New World Junín induces an autophagic, proviral response with decreased viral replication in Atg5 and Beclin 1 knockout and knockdowns, both of which are critical components of the autophagic pathway [66].

Through such mechanisms, the Z protein contributes to the virus's ability to evade detection and suppression by the innate immune system, facilitating the establishment of infection and the potential for viral dissemination.

## RNA-dependent RNA polymerase L

LASV L protein serves several distinct roles during viral infection: replication of the viral genomic RNA, generation of viral antigenomes, and the synthesis and capping of viral mRNA. Due to the ambisense orientation of LASV genes, the L and NP genes, which are encoded in the viral genome in an anti-sense orientation, are transcribed by the L protein directly from the viral genome.

As previously described, the RdRP encoded by the L protein is responsible for the synthesis of RNA complementary to the viral RNA template. All segmented negative sense RNA viruses in the subphylum Polyploviricotina have highly conserved RdRP motifs involved in RdRP catalytic activity, metal ion binding, adenosine triphosphate (ATP) binding, and an EF/YXS motif important in cap-snatching [63, 67–73]. L protein from a related segmented negative-strand RNA virus, thrombocytopenia syndrome virus, has been found to interact with mRNA from the evolutionarily-conserved host WNT signal transduction pathway through cap-snatching [74]. This implies a conserved replication method among segmented negative-strand RNA viruses, including LASV, and potential antiviral targets. Some of the large genetic diversity of LASV isolates can be explained by the flexibility of substitutions allowed in the linking sequences found between the conserved motifs in the L-core domain [75, 76].

The LASV L protein contains a putative N-terminal endonuclease domain that mediates cap-snatching, a core domain with 6 amino acid motifs that are highly conserved among other viruses in the Bunyavirales order and among Orthomyxovirus RdRPs, and a C-terminal domain that is poorly characterized but seems to play an important role in protein–protein interactions and transcription, potentially through putative cap binding motifs [63, 67, 77, 78] (Fig. 3B). Full-length L protein does not demonstrate intrinsic endonuclease activity, and the catalyst for cap-snatching has yet to be determined [63, 79], although this interaction is hypothesized to be mediated by the Z protein [80]. In the pre-initiation phase of RNA synthesis by the L protein, its endonuclease activity is uninhibited; whereas in the early elongation phase its activity is inhibited by two L protein motifs that bind to the active site [79]. Metal chelators, including diketo-acids and N-hydroxyisoquinoline-1,3-diones, have been shown to inhibit LCMV L protein’s cap-dependent endonuclease activity, indicating a dependence on metal ions [81, 82].

Molecular analysis of the protein–protein interactions of the full-length L protein has been hindered because plasmids containing full-length L cannot be easily propagated in *Escherichia coli*. This leads to challenges in rapidly generating mutational variants of LASV L in vitro [83]. OW arenavirus reassortants among LASV, MOPV, and LCMV strains have strongly linked pathogenicity to the LASV and LCMV L-segments [84–88]. Recently, strain-based differences in the pathogenicity of LASV in a guinea pig model also mapped to the L-gene [89]. LASV replication efficiency measured by viremia or viral titer in organs correlates with pathogenesis, a phenomenon which is heavily supported by the therapeutic benefit of antiviral Ribavirin in reducing viral titers [90, 91]. However, the relationship between efficiency of replication, L-gene function, and the non-coding regions of the L-segment in pathogenesis is poorly understood.

## Nucleoprotein

NP is the most abundant viral protein in both infected cells and virions [92]. The NPs of all arenaviruses (with the exception of TCRV, the prototypical NW arenavirus) display a high degree of sequence and structural homology [93, 94]. Starting with the N-terminus, which is comprised of a proposed multimerization domain, the general NP structure contains an RNA-binding domain and is followed by an exonuclease domain near the C-terminus [93, 95] (Fig. 3C). When expressed in mammalian cells, LASV NP forms trimers that are essential for viral transcription and replication [96]. NP and L associate with vRNA to form the RNP; these two proteins are the minimal trans-acting factors necessary for replication of LASV vRNA [97, 98]. The amount of NP expressed in cells directly corresponds to the reporter activity of a LCMV minigenome (MG), highlighting its central role in transcription and regulation [99]. Reducing the levels of available LASV NP in cells by pretreatment with small interfering RNA (siRNA) results in decreased negative-sense viral replication and enhanced interferon responses [100].

The N-domain of the NP monomer contains a crevice that mediates binding to vRNA and plays a role in antigenome synthesis [101]. The first reported crystal structures described NP as a homotrimer, but this configuration prevents vRNA binding because the gate allowing for RNA access is locked in a closed position [93, 101]. Despite this, the ability to multimerize is critical for replication and translation of the vRNA genome, and promotes interaction of NP with the viral RdRP L protein [101]. In the presence of RNA, the homotrimer monomerizes spontaneously through a global conformation change in which NP’s C-terminal domain swings out away from the N-terminus to expose the RNA-binding pocket [39, 102]. NP can additionally bind Z, regardless of either RNA binding or whether it is in monomeric or trimeric form. However, Z’s binding affinity decreases at lower pH, which may play a role in the release of RNP complexes during fusion within endosomes [39].

Studies done in LCMV have shown that GP1 and NP colocalization with host proteins coat protein I (COP-I) and adaptor protein 4 (AP-4) complexes are required for LCMV assembly and egress [103]. Silencing COPA and COPB1, subunits of the COP-I complex, result in 50% reduction of recombinant LCMV expression. The authors suggest that NP is responsible for the redistribution of AP-4 subunit component AP4E1 from the Golgi to the cytoplasm, affecting its role in secretory and endocytic pathways and ultimately LCMV egress. Similarly, in another study, silencing of the tailless complex polypeptide 1 ring complex (TRiC/CCT) inhibited LCMV replication in vitro by interacting with NP as a substrate [104].

Host immune responses to arenaviruses are heavily dependent on detection of virus components by the pattern recognition receptors RIG-I, melanoma differentiation-associated protein 5 (MDA5), protein kinase R (PKR), and Toll-Like Receptors 7 (TLR7) and 9 (TLR9), in each case activating type-I IFN signaling [105, 106]. However, the kinetics of viral sensing and IFN induction are highly nuanced, host-cell type specific, and virus strain dependent [105, 107, 108]. Similar to other arenavirus NPs, LASV NP has potent anti-type I IFN activity which maps to the exoribonuclease catalytic pocket within the C-domain of the protein [93, 109]. LCMV vRNA is recognized by cellular RIG-I and MDA5, leading to IFN regulatory factor 3 (IRF3) or 7 (IRF7)-mediated production of type-I IFNs [109, 110]. Interestingly, PKR binds to and is strongly activated by pathogenic NW arenaviruses Junín (JUNV) and Machupo (MACV), but not by LASV [111–113].

The LASV NP exoribonuclease domain shares sequence and structural homology with the ubiquitous DEDDH superfamily of exonucleases, including the hallmark motif (Asp-Glu-Asp-Asp with a histidine in close apposition) that mediates the specific 3’ to 5’ degradation of double-stranded RNA (dsRNA) and is thought to be essential for LASV NP’s immunosuppressive activities [114]. The exoribonuclease enzymatic function of NP degrades viral dsRNA, preventing its recognition by RIG-I and MDA5 [93, 114, 115]. This degradation activity is limited to OW arenaviruses, as infection with NW JUNV or MACV results in accumulation of viral dsRNA [113, 116]. Recently, it has been reported that in the absence of the RdRP L protein, the exoribonuclease catalytic function of NP is necessary but not sufficient to prevent RIG-I and PKR recognition of vRNA during LASV infection [113]. An additional layer of complexity of LASV type-I IFN antagonism is evidenced by the high-affinity interaction of the C-terminus domain of LASV NP directly with IRF 3/7 kinases I-kappa-B kinase epsilon (IKKε), a master regulator of the NF-κB inflammatory signaling pathway. This interaction occurs independent of NP’s exoribonuclease catalytic activity, and blocks the autophosphorylation and downstream signaling of IKKε [117].

LASV NP also interacts with DDX3, a DEAD (Asp-Glu-Ala-Asp)-box ATP-dependent RNA helicase, to facilitate both virus replication and suppression of the type-I IFN response [118–121]. At early timepoints of viral infection, host cells that produce recombinant DDX3 proteins that lack either ATPase or helicase catalytic activity show reduced expression of a LCMV MG reporter for virus replication [118]. The presence of DDX3 during LASV infection increases infectivity by suppressing type I IFN signaling [118]. For LASV, NP has been hypothesized to modulate a number of interactions to prevent establishment of an antiviral state. Some of these interactions include NP with RIG-I/MDA5, the RIG-I/MDA5 and vRNA complex with IKKε, NP directly with DDX3, and DDX3 with IKKε, although the exact mechanisms have yet to be determined [118]. Additionally, reports of LCMV NP-mediated inhibition of the immune regulator NF-κB in an IKKε and TANK-binding kinase 1 (TBK1) dependent manner are consistent with these findings [122].

The cleavage of pro-caspases to mature caspases is a key step in the initiation of apoptosis [123]. JUNV NP contains two caspase cleavage recognition sites (DVKD and QEHD) that function as decoys, leading to decreased apoptosis of infected cells [124]. Similar NP cleavage products have been identified in LASV virions, causing speculation that LASV uses the same mechanism to abrogate apoptosis as an additional immune evasion strategy [125]. Furthermore, it is possible that NP cleavage products serve additional functions in the viral life cycle [124].

## Glycoprotein

The LASV GPC gene produces a protein that is cleaved by host proteases into GP1, which is the main surface exposed component of mature GPC trimers that coat the exterior of virions and mediate canonical receptor binding; GP2, which mediates fusion of host and viral cell membranes; and a stable signal peptide (SSP) [126, 127] (Fig. 3D). On the surface of virions, GPC exists as a trimer of heterotrimers of non-covalently linked GP1, GP2 and SSP [126]. GP2 is a class I viral fusion protein which is inserted into the lysosomal membrane, undergoes a conformational change to bridge the virion and cell membrane, and is left in a characteristic six-helix bundle as confirmed by the post-fusion crystal structure [128–131]. SSPs are highly conserved among all arenaviruses, and are larger than canonical signal peptides [126, 132, 133]. Once produced, GPC is transported to the endoplasmic reticulum (ER) and Golgi apparatus for processing, folding, and post-translational modification. Following co-translational cleavage of SSP, GPC is processed by subtilisin kexin isoenzyme‐1/site 1 protease (SKI‐1/S1P) into GP1, which is heavily glycosylated, and GP2 [133–135]. Glycosylation of the GPC has been noted to play a major role in determining arenavirus virulence and pathogenicity. These modifications and interactions have been thoroughly reviewed by Gorzkiewicz et al. [136]. Unlike Filovirus glycoproteins, LASV GPC is not associated with direct pathology in terms of causing vascular leakage [137–139]. Detailed mutational analysis of the SSP of LCMV revealed that it is essential not only for the translocation of GPC, but also for GPC processing, cell surface transport of GP1 and GP2, formation of virions, and fusion [132]. However, the role and localization of SSP during entry and fusion has yet to be described [140].

α-dystroglycan (αDG), a ubiquitous cell surface glycoprotein, is the primary receptor for OW arenaviruses including LASV [141, 142]. However, αDG-independent entry has also been observed, with involvement from TAM family kinases AXL or Tyro3, C-type lectins, dendritic cell (DC)-specific intracellular adhesion molecule 3 (ICAM-3)-grabbing nonintegrins (DC-SIGN), liver and lymph node sinusoidal endothelial calcium-dependent lectin (LSECtin), and lysosomal sialomucin CD164, which suggests that the complexities of LASV entry have yet to be fully described [127, 143–146]. In the case that glycosylation is altered in a way that prevents viral entry via canonical receptors, arenaviruses have been shown to find alternative receptors for entry, such as heparan sulfate proteoglycans [147]. Following receptor binding, LASV is internalized into host cells through clathrin-independent macropinocytosis [148]. Interestingly, the well-characterized entry mechanism appears to be unique among viruses utilizing micropinocytosis, as classical disruption of the cell membrane does not occur [148]. Acidification of the macropinocytotic vesicle during lysosomal maturation causes a conformational change that leads to a receptor switch and allows GP1 to bind lysosomal-associated membrane protein 1 (LAMP1), which precedes dissociation of GP1 from the GPC complex prior to fusion [140, 149, 150].

The recent publication of a neutralizing antibody (nAb)-bound surface GPC crystal structure has provided insight into the humoral response to LASV [151]. Extensive glycosylation of GP1 shields much of the ectodomain from antibody recognition [126, 127]. However, nAbs that recognize unshielded portions of the quaternary structure, or prevent conformational changes that occur during entry and fusion, may arise in response to virus perturbation [126, 127, 151]. Prefusion-stabilized trimeric GPCs of phylogenetically distinct LASV lineages were characterized by Perrett et al. and found to display similar structural features despite extensive sequence diversity [152].

To add even more complexity to the current model requiring surface receptors for viral entry, recombinant LCMV has been shown to infect cells via intercellular connecting tunneling nanotubes (TNT) [103]. NP, GP1, and Z were all shown to localize with the TNT-like structures during infection, and blocking the classical egress pathway post-infection causes viral transmission via TNT. Further investigation into this alternative pathway is necessary to determine its importance in viral infection and its relation to pathogenesis.

## Non-coding regions

The non-coding regions of the LASV genome, namely the 5’ and 3’ untranslated regions (UTRs) and the intergenic region (IGR) of each RNA segment, play key roles in the viral life cycle, and therefore cannot be ignored when discussing LASV pathogenicity. The absence or truncation of the IGR interferes with the ability of the virus to generate infectious virions for multiple OW and NW arenaviruses [153–155]. The IGR functions as a transcriptional terminator that allows the generation of mRNA from vRNA [153]. While the viral genomic and antigenomic RNA have independent mRNA activity, it is significantly reduced compared to the capped, non-polyadenylated mRNA produced from transcriptional termination by the IGR [156]. While it has yet to be demonstrated experimentally, the IGR may interact with the 3’ UTR to enhance the translation of non-polyadenylated mRNA, similar to what has been observed in Bunyaviruses [157–159].

For LCMV, the function of the S-IGR and L-IGR are distinct, as evidenced by the in vivo attenuation of mutants in which the L-IGR is replaced by the S-IGR [153, 159]. The S-IGR of LCMV has a substantially different sequence, but similar structure, as compared to the S-IGR of LASV. Swapping the S-IGR of LCMV for that of LASV does not significantly reduce viral gene expression, indicating that the structure of the IGR is may be more important than the sequence in determining its function [160]. The IGR may also interact with Z to facilitate the incorporation of vRNA into virions, although the mechanism of this interaction has yet to be elucidated [156]. Replacing the IGR of LCMV, even with distantly related or synthetic IGR structure-like RNA, still generated functional viral particles, suggesting the interaction of the IGR and Z is not specific [159, 160].

The 5’ UTR and 3’ UTR of both the L and S segments of all arenaviruses contain 19 nucleotide (nt) sequences which are nearly completely complementary and form panhandle structures. Mutational analysis in the LASV UTR demonstrated that both the structure and the sequence of the first 12 nt pairs are strict requirements for minigenome replication, while the last 7 nt pairs enhance promoter function in a non-sequence specific manner [161, 162]. In LCMV, this equivalent translation-promoting sequence has been isolated to a stem-loop structure present in the last 10 nt of the 3’ UTR, allowing for increased virus replication [163]. The L RdRP uses a prime-and-realign mechanism, where polymerase binding leads to a slip that results in a non-templated 5’ guanine triphosphate (5’pppG) [164, 165]. The single nt 5’pppG unpaired overhang, combined with single nt mismatches in the panhandle structure, hinders vRNA recognition by RIG-I and MDA5, therefore allowing the virus to evade host anti-viral responses [164, 166]. Similarly, partial deletions and point mutations in the 5’ internal noncoding regions next to the terminal 19-nt sequences reduced recombinant TCRV infection in vivo [167]. Recently, the involvement of UTR sequences of the LCMV S-segment outside of the first 19 nt were found to be critical to virulence in vivo; however, the mechanistic contribution to pathogenicity has yet to be determined [168].

## Pathogenesis resulting directly from host responses

Lassa fever fails to show some classical signs of viral hemorrhagic fevers, including cellular necrosis, leukopenia (LASV instead causes neutrophil-based leukocytosis), trigger of disseminated intravascular coagulation, and a cytokine storm [150–152]. While LASV can cause coagulopathies, the mechanisms which disrupt endothelial function are cryptic, and may be more related to immune dysregulation leading to hepatic necrosis and renal dysfunction than any direct LASV-mediated effect [172–176]. LF hepatic necrosis is characterized by the infiltration of Kupffer cells (hepatic macrophages) along with the absence of T cells and antigen presenting cells [177].

LASV infects myeloid cells, including macrophages and dendritic cells (DCs); and while it does not reduce the viability of these cells, they do not produce a significant cytokine response, stimulate T cells, show signs of activation of DCs, or trigger maturation of monocytes [178, 179]. In contrast, MOPV strongly activates DCs and elicits robust CD4^+^ and CD8^+^ T cell immunity [180]. LASV readily infects endothelial cells and inhibits production of proinflammatory cytokines including interleukin 8 (IL-8) and tumor necrosis factor α (TNF-α), a phenomenon which may precipitate the failure to activate myeloid cells [138]. Low cytokine levels of IL-8 and inducible protein 10 (IP-10) are biomarkers associated with fatal outcomes in LASV patients [181]. A mechanism has been proposed that varying levels of TLR2 activation by arenaviruses in the endosome affects the outcome of infection based on the resulting level of proinflammatory cytokine signaling [108, 182–184].

Proinflammatory cytokine signaling is needed to generate CD8^+^ cytotoxic T cells, the main effectors responsible for cell-mediated immunity (CMI) associated with the resolution of LASV infection [182, 185–188]. This LASV-specific CD8^+^ response is detectable with restimulation of samples from surviving patients that were infected over 10 years after initial evaluation [189]. CMI responses aided by CD4^+^ T cells specific for both NP and GP2 have been reported [190, 191]. Specific NP and GPC epitopes that are recognized by HLA in the generation of protective CMI have been described [192–194]. However, peptide vaccination of LCMV-immune mice with LCMV-specific epitopes caused rapid and fatal CD8^+^ T-cell mediated hyper-immunity [195].

LASV is the leading cause of non-congenital hearing loss in West Africa [16, 196, 197]. Cynomolgus macaques that survived LASV challenge and developed sensorineural hearing loss had systemic vasculitis and display numerous gross pathological signs of autoimmune disease [198]. STAT-1 knockout mice, which are not responsive to type I or type II IFN signaling, are susceptible to LASV and develop hearing loss following infection with non-pathogenic LASV isolates [196, 199]. In studies with ML29, a highly-attenuated arenavirus reassortment model including the S segment of LASV Josiah and L segment of MOPV, Reyna et al. infected STAT-1 knockout mice and determined that hearing loss in this model occurs through a CD8^+^ T cell-independent mechanism after observing hearing loss despite T cell depletion [200].

The isolation of virus from patient samples with high titer of IgG demonstrates that neutralizing antibody (nAb) likely does not play a significant role in the natural resolution of LASV infection [201]. Most neutralizing responses are directed towards GP1 or the GP1/2 interface; it is possible that the high level of glycosylation of GP2 on the surface of virions is not conducive to the generation of GP2 specific nAbs [202]. Passive transfer of serum from guinea pigs that previously had been successfully vaccinated against fatal LASV challenge did not confer protection, although a follow-up study demonstrated that protection could be conferred if the plasma contained high titer of nAb [203–205]. Hyperimmune antibodies generated from immunized rabbits were able to protect interferon alpha receptor knockout mice from lethal LASV challenge, however nAb titers were not determined precluding correlation of neutralizing activity to treatment success [206]. Early clinical trials of convalescent plasma in LASV patients had mixed results, despite evidence that it is beneficial if given early in infection to rhesus macaques [90, 91, 207]. However, with advances in monoclonal antibody (mAb) technology, it has been suggested that highly neutralizing human mAbs may be a useful therapeutic, which is supported by their ability to rescue cynomolgus macaques from the late stages of lethal infection [208, 209]. Recent studies highlight progress in mAb drug development using cocktails derived from human LASV survivors to protect non-human primates from challenge with LASV from clades II, III and IV, although efficacy was limited against challenge with a clade VII isolate from Togo [210–212].

## Conclusions

Lassa virus has a complex interplay of host-virus interactions that are crucial for its lifecycle, pathogenesis, and battle with the host's immune system. The Z protein, serving as a matrix protein, is central to virus assembly and budding, and it interacts with components of the host's cellular machinery, such as the ESCRT pathway, to facilitate viral egress. It also plays a role in modulating the host's innate immune response, potentially by interacting with cellular proteins like PML. The L protein, functioning as the viral RNA-dependent RNA polymerase, is essential for both replication of the viral genome and transcription of viral mRNAs, requiring interaction with the NP protein for efficient viral RNA synthesis. The NP protein, beyond its role in encapsulating viral RNA, has been implicated in suppressing the host's interferon response via interactions with RIG-I/MDA5, IKKε, and DDX3 among others. The GPC, responsible for receptor binding and cell entry, undergoes proteolytic processing to produce the GP1 and GP2 subunits, which mediate attachment to the cellular receptor α-DG and subsequent fusion of cellular and viral membranes, respectively. These host-virus interactions underscore the complexity of LF pathogenesis as LASV hijacks host cellular processes for replication and to evade immune detection. Disrupting these interactions could prove to be an effective strategy for therapeutic intervention against LF.

While minigenome systems and the rescue of recombinant viruses has vastly increased the understanding of LASV molecular pathogenesis, there are many questions that remain. LCMV provides a convenient model virus that can be utilized outside of maximum containment labs; however, care must be taken when interpreting the results of these experiments in terms of extrapolation to the molecular mechanisms of LASV to cause LF. Using reverse genetics approaches to compare highly studied LASV isolates, non-pathogenic strains of LASV as well as the closely related MOPV, and recent clinical isolates with varying levels of pathogenicity will provide insight into the molecular determinants of LF. Furthermore, it appears that several key aspects of LASV pathology are mediated by the host response to the virus rather than by viral factors, highlighting the need for advanced in vivo models of arenavirus hemorrhagic fever disease.

## Data Availability

No datasets were generated or analysed during the current study.
